# MiR-29a Curbs Hepatocellular Carcinoma Incidence via Targeting of *HIF-1α* and *ANGPT2*

**DOI:** 10.3390/ijms23031636

**Published:** 2022-01-31

**Authors:** Ying-Hsien Huang, Wei-Shiung Lian, Feng-Sheng Wang, Pei-Wen Wang, Hung-Yu Lin, Ming-Chao Tsai, Ya-Ling Yang

**Affiliations:** 1 Department of Pediatrics, Kaohsiung Chang Gung Memorial Hospital Chang, Kaohsiung 833, Taiwan; yhhuang123@yahoo.com.tw; 2 College of Medicine, Chang Gung University, Taoyuan 333, Taiwan; 3 Center for Mitochondrial Research and Medicine, Kaohsiung Chang Gung Memorial Hospital, Kaohsiung 833, Taiwan; lianws@gmail.com (W.-S.L.); wangfs@ms33.hinet.net (F.-S.W.); wangpw@cgmh.org.tw (P.-W.W.); 4 Core Laboratory for Phenomics & Diagnostics, Department of Medical Research, Kaohsiung Chang Gung Memorial Hospital and Chang Gung University College of Medicine, Kaohsiung 833, Taiwan; 5 Department of Internal Medicine, Kaohsiung Chang Gung Memorial Hospital and Chang Gung University College of Medicine, Kaohsiung 833, Taiwan; 6 Research Assistant Center, Show Chwan Memorial Hospital, Changhua 500, Taiwan; linhungyu700218@gmail.com; 7 Division of Hepato-Gastroenterology, Department of Internal Medicine, Kaohsiung Chang Gung Memorial Hospital and Chang Gung University College of Medicine, Kaohsiung 833, Taiwan; tony0779@gmail.com; 8 Department of Anesthesiology, Kaohsiung Chang Gung Memorial Hospital and Chang Gung University College of Medicine, Kaohsiung 833, Taiwan

**Keywords:** miR-29a, hepatocellular carcinoma, HIF-1α, ANGPT2

## Abstract

A high-fat diet is responsible for hepatic fat accumulation that sustains chronic liver damage and increases the risks of steatosis and hepatocellular carcinoma (HCC). MicroRNA-29a (miR-29a), a key regulator of cellular behaviors, is present in anti-fibrosis and modulator tumorigenesis. However, the increased transparency of the correlation between miR-29a and the progression of human HCC is still further investigated. In this study, we predicted HIF-1α and ANGPT2 as regulators of HCC by the OncoMir cancer database and showed a strong positive correlation with HIF-1α and ANGPT2 gene expression in HCC patients. Mice fed the western diet (WD) while administered CCl4 for 25 weeks induced chronic liver damage and higher HCC incidence than without fed WD mice. HCC section staining revealed signaling upregulation in ki67, severe fibrosis, and steatosis in WD and CCl4 mice and detected Col3a1 gene expressions. HCC tissues significantly attenuated miR-29a but increased in HIF-1α, ANGPT2, Lox, Loxl2, and VEGFA expression. Luciferase activity analysis confirms that miR-29a specific binding 3′UTR of HIF-1α and ANGPT2 to repress expression. In summary, miR-29a control HIF-1α and ANGPT2 signaling in HCC formation. This study insight into a novel molecular pathway by which miR-29a targeting HIF-1α and ANGPT2 counteracts the incidence of HCC development.

## 1. Introduction

Hepatocellular carcinoma (HCC) is considered the sixth most common cancer and the second principal detriment of cancer-related deaths worldwide, accounting for approximately 841,000 new cases and 782,000 deaths every year [1,2]. Risk factors include viral hepatitis B, hepatitis C, alcoholic fatty liver, non-alcoholic fatty liver disease (NAFLD), and non-alcoholic steatohepatitis (NASH) are ultimately the causes of liver fibrosis and cirrhosis, and nearly 70–80% will convert into HCC patients [3,4]. Solid tumors have a feature on insufficient oxygen supply and create hypoxia microenvironment to induce many abnormalities with prognostic consequences. Hypoxic is a hallmark of cancer and a key factor for angiogenesis and dysregulation of the immune response [5]. HCC exhibited upregulation of hypoxia-inducible factors (HIFs) and mediated neovascular and lymphatic vessel formation process to create an appropriate niche, allowing tumor progress, multiplying, and disorganized architecture. Emerging pieces of evidence suggest that antiangiogenic drugs with novel immuno-oncology drugs or other drugs with novel mechanisms of action would be an essential way to explore diverse therapeutic strategies [6].

MicroRNAs (MiRs) are approximately 22 nucleotides in length, short non-coding RNAs that function in a pathway-centric manner by targeting multiple genes and are potential therapeutic targets for HCC [7]. Previously, we have illustrated the biological role of miR-29a in the context of hepatic disorders, such as liver fibrosis [8,9,10], NAFLD [11,12,13], and NASH [14]. Mounting evidence has unveiled that NAFLD and NASH connote progressive liver injury leading to liver cirrhosis and hepatocellular carcinoma [15]. We employ the Gene Expression Omnibus (GEO) dataset corroborated the differential expression and diagnostic value of miR-29a [16]. Moreover, we have previously uncovered an anti-HCC effect of miR-29a via comprehensively suppressing the expression of lysyl oxidase family members, Lox, Loxl2, and vascular endothelial growth factor A (VEGFA) [16]. VEGFA plays a significant role in angiogenesis, while angiopoietin (ANGPT) growth factors, such as ANGPT1 and ANGPT2, regulate vascular stabilization and remodeling during angiogenesis [17].

The transcription factor hypoxia-inducible factor-1 alpha (HIF-1α) has been characterized as the master regulator of cellular adaption to hypoxia in tumorigenesis microenvironment [18], which acts to promote various gene expressions, including angiogenic inducer VEGFs, VEGFRs, and ANGPTs [19]. Low oxygen tension-dependent HIF-1α and constitutively expressed HIF-1β form active HIF-1 [20]. HIF-1α exerts transcriptional activity by directly binding to downstream target genes, like VEGF and VEGFR1 gene promoters at the hypoxia-responsive elements (HREs) site and induce the transcription of VEGFA and VEGFR1 genes. Excessive HIF-1α dominates the maintenance of the tumor microenvironment, and studies have shown that inhibiting HIF-1α-binding protein CDK5 affected HIF-1α activity could effectively suppress the angiogenesis of tumorigenic [21].

Hypoxia is a hallmark of solid cancers, especially HCC mediating metabolic reprogramming in drug resistance in HCC [22]. Inasmuch as Lin and Wei et al., respective mentioned that HIF-1α was associated with the prognostic value of patients with HCC following trans-arterial chemoembolization (TACE) [23,24]. Concerning the molecular basis of miR-29a at the post-transcriptional level, one bioinformatic survey predicted that miR-29a targets the 3′-untranslated region (3′-UTR) of HIF-1α and ANGPT2. As miR-29a possesses an emerging role in HCC, whether it is implicated in the regulation of HIF-1α in the context of HCC remained unanswered. We demonstrate that miR-29a regulates the expression of HIF-1α and ANGPT2 in vivo and in vitro, offering novel insights into the miR-29a -involved chronic liver disease in the developing of a practical diagnostic/prognostic panel for HCC.

## 2. Results

### 2.1. MIR-29a Is a Significant Suppressor of HIF1A and ANGPT2 in HCC

**Figure 1:**
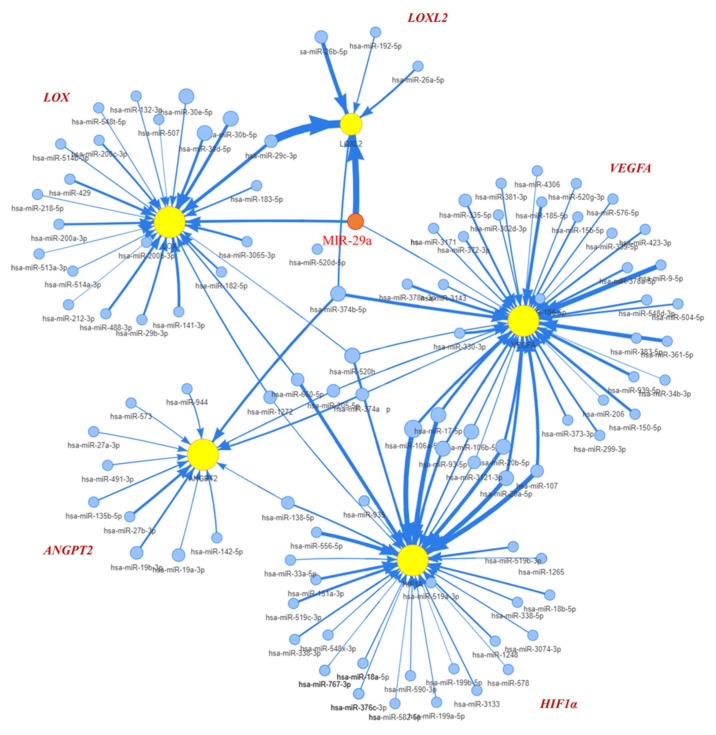
Interpretation of miR-29a regulates HIF1A and ANGPT2 expression. The OncoMir database analysis delineates the miR-29a interaction edge that suppressed the expression of HIF1A, ANGPT2, and VEGFA.

**Figure 2:**
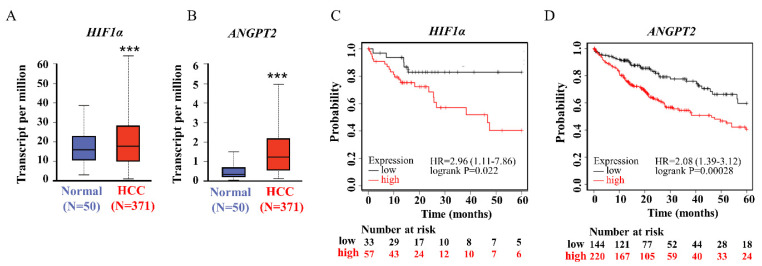
The HIF1A and ANGPT2 expression were committed on HCC patients. (**A**,**B**) The HIF–1 and ANGPT2 expression levels in human HCC and normal tissue by regained of TCGA cohort database. (**C**,**D**) The HIF–1 and ANGPT2 expression levels are significantly correlated with the probability of survival in HCC patients by Kaplan–Meier survival analysis. *** *p*-value < 0.001 was comparative between the normal and HCC group.

Given previews results, we delineated the contribution of miR-29a as a principal element that affected pathogenesis of chronic liver disease and HCC [16]. TargetScan (version 7.2) and the GSCALite platform was used to prognostic miR-29a regulated tracks and define the edge of the gene network (Figure 1). Prediction networks showed HIF1A and ANGPT2 as candidate target genes of miR-29a. Thus, we examined HIF1A and ANGPT2 in HCC patients and matched normal data to evaluate their roles in HCC progression. HIF1A and ANGPT2 gene expression were characterized by managing the bioinformatics web-based platform UALCAN. The mRNA expression data were obtained from RNA-seq profiles and generated from the TCGA (The Cancer Genome Atlas) datasets. The results showed that, compared with normal clinical patients, the expression of HIF1A and ANGPT2 in HCC was upregulated (Figure 2A,B), and was correlated with decreased HCC patient survival rate by the Kaplan-Meier method analysis.

### 2.2. Western Diet (WD) Combine Carbon Tetrachloride (CCl4) Treated Promote Chronic Liver Disease and Cancer Formation

**Figure 3:**
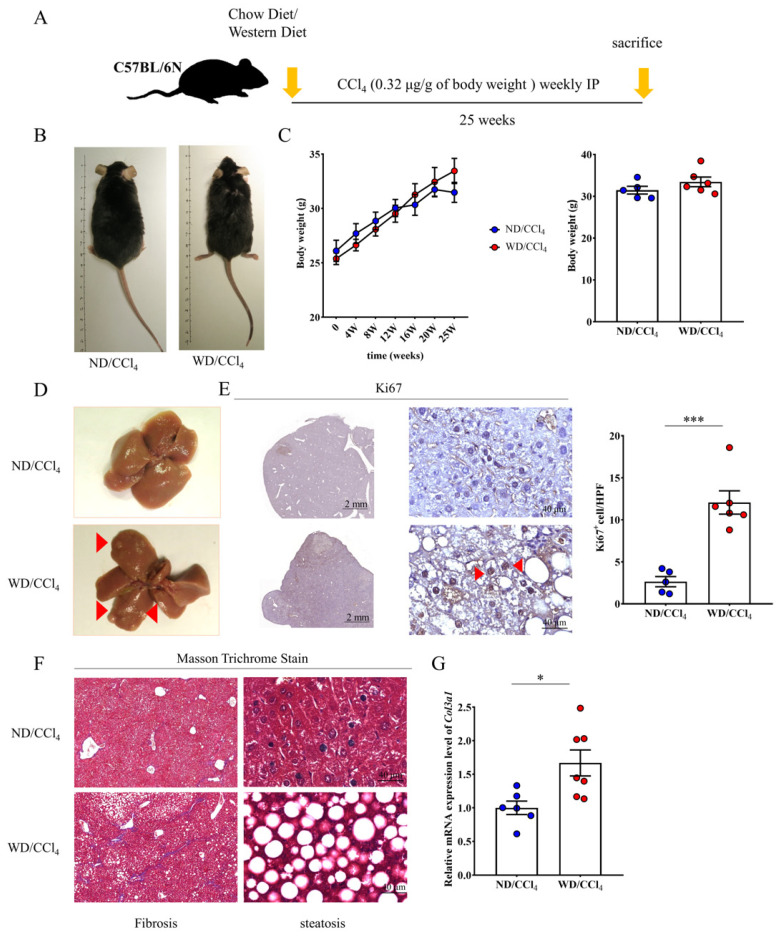
Western diet (WD) and carbon tetrachloride (CCl4) treatment promote HCC formation in the mouse. (**A**) Schematic diagram of establishing a mouse HCC process. (**B**,**C**) Observation of appearance and body shape of mice in HCC and the range of weight change in time course. (**D**) Representative liver tissue macroscopic images and WD/CCl4 group indicate the number of red arrow numbers. (**E**) Liver tissue section of the WD/CCl4 group showed ki67 positive immunostaining. Scale bar, 2mm, and 40μm indicated low and high magnificently. (**F**) Masson trichrome stained livers presence of hepatic fibrosis and steatohepatitis. Scale bar, 40 μm. (**G**) Col3a1 gene expression elevated in WD/CCl4 treated group. Data per group are expressed as mean ± SEM calculated from six to seven mice. * *p*-value < 0.05; *** *p*-value < 0.0001 between groups.

To generate a clinical comparative animal model and accelerated progress of HCC, we followed administration procedures described by Tsuchida et al. [25], which consists of multiple treated CCl4 with Western diet feed as the WD/CCl4 group (Figure 3A). Feed WD was shown to enhance and accelerate the roughness of fur, but there was no significant difference in body weight compared with the normal diet (ND) plus CCl4 group (Figure 3B,C). A significant discrepancy in liver pathology was observed between ND/CCl4 and WD/CCl4 groups. After 25 weeks of WD/CCl4 treatment, nodules were obtained in the liver tissue of mice, indicating that hepatocellular carcinoma (HCC) was induced and successfully established an animal disease model (Figure 3D). To test whether cancer-related gene ki67 involve in HCC growth and proliferation. We performed immunohistological analysis, demonstrated that liver tissue from the WD/CCl4 group displayed strong signaling of ki67 and quantified positively stained cells (Figure 3E). Masson staining showed that the WD/CCl4 group also resulted in severe liver fibrosis and infiltration of inflammatory cells under the disordered lobular structure (Figure 3F). In addition, we further performed qRT-PCR to detect the expression level of Col3al, which confirmed that the liver tissue of the WD/CCL4 mice had excessive collagen deposition in liver tissue compared to ND/CCl4 mice (Figure 3G).

### 2.3. WD/CCl4 Treated Intervention miR-29a Expression and Promoted Tumorigenesis Signaling in Liver Tissue

**Figure 4:**
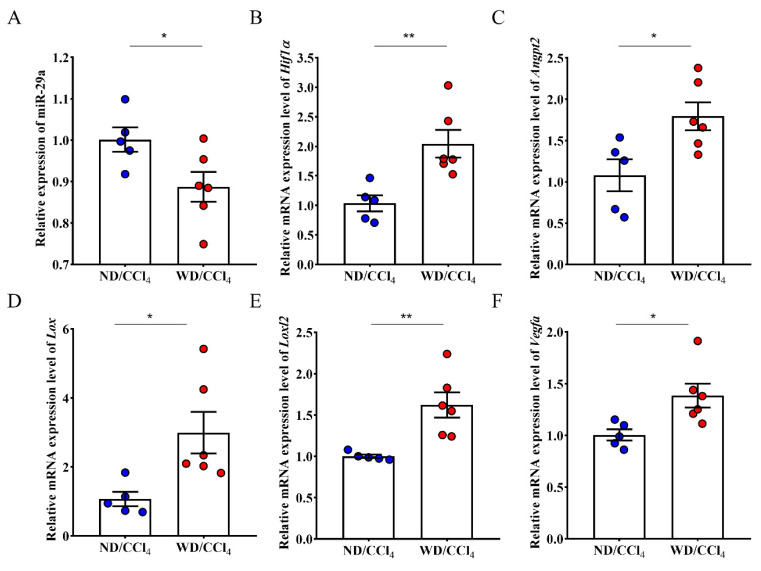
Differential expression of tumorigenesis associated genes expression in liver tissue of ND/CCl4 and WD/CCl4 group. (**A**) The expression of miR-29a in the WD/CCl4 treatment group was significantly reduced, and (**B**–**F**) five related genes were significantly increased. Data per group are expressed as mean ± SEM calculated from five to six mice. * *p*-value < 0.05; ** *p*-value < 0.001 between groups.

Furthermore, miR-29a decreased expression in high-risk HCC using WD/CCl4-treated mice (Figure 4A). Expanding evidence has revealed that miR-29a is indispensable in chronic liver disease [26]. We uncovered that liver tissue of mice treated with WD/CCl4 upregulated the expression of HIF-1α and Angpt2 (Figure 4B,C). This finding is consistent with liver cancer patients’ bioinformatics analysis and regulatory network, whereas Lox, Loxl2, and Vegfα mRNA expression also increased in WD/CCl4 mice rather than ND/CCl4 mice (Figure 4D–F).

### 2.4. miR-29a Targeted the 3′-UTR of HIF-1a and ANGPT2

**Figure 5:**
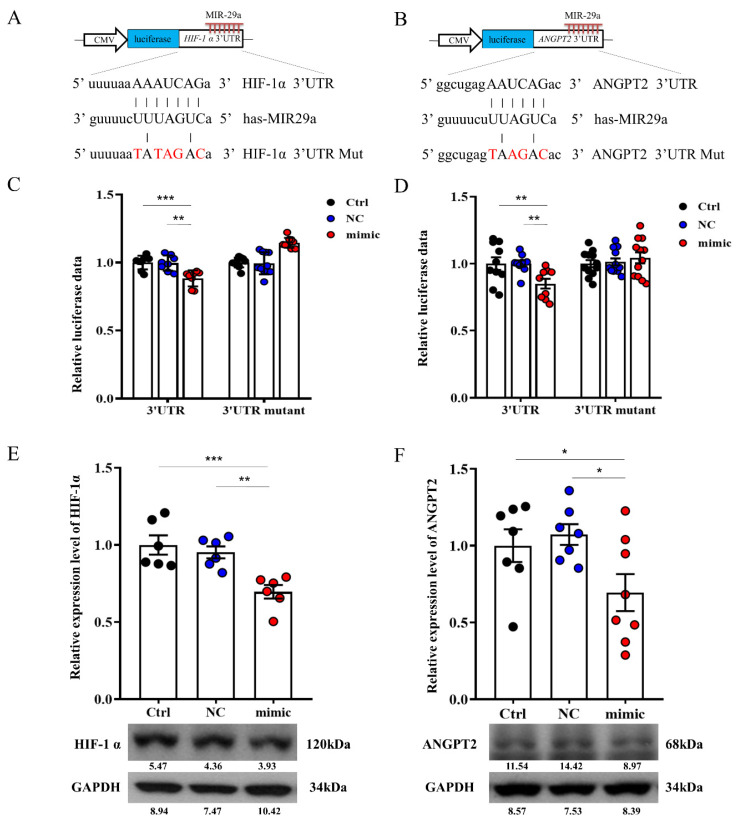
Effect of miR-29a signaling on HIF-1a and ANGPT2 in hepatocytes. (**A**,**B**) Illustrated 3’UTR sequences of HIF-1a and ANGPT2 are specific for miR-29a binding. Overexpression of miR-29a decreased 3’UTR luciferase report activity (**C**,**D**) and protein activities (**E**,**F**) of HIF-1a and ANGPT2. Knocking down by mutant sequence of HIF-1a and ANGPT2 are diminished miR-29a-3′-UTR luciferase reporter activity. Data per group are expressed as mean ± SEM. * *p*-value < 0.05; ** *p*-value < 0.01, *** *p*-value < 0.001 between groups.

As predicted by bioinformatics (miRBase 22.1) show HIF-1a and ANGPT2 putative target of miR-29a. We constructed luciferase reporters for control, and the 3-base pair mutated 3′-UTR of HIF-1a and ANGPT2 (Figure 5A,B) that deciphered how miR-29a affected specific areas gene expression in HepG2 cells. Notably, increased miR-29a significantly decreased luciferase reporter activity of 3′-UTR of HIF-1a and ANGPT2 (Figure 5C,D), whereas miR-29a-mimic also attenuated protein expression of HIF-1a and ANGPT2 (Figure 5E,F).

## 3. Discussion

Hepatocellular carcinoma (HCC) is a common malignant liver disease, which accounts for one of the leading cause of mortality globally [2]. Clinical diagnosis of various liver diseases, including chronic viral and C, alcoholic liver disease, NAFLD and NASH, etc., all increase the probability of HCC [27,28]. While accumulating studies reveal that dysfunctional hepatic cells, oxidative stress, and immune dysregulation accelerate HCC development [29,30], little is known about how chronic liver disorders shift into HCC. Clarifying the underlying molecular mechanisms of the progression of HCC is essential for determining novel therapeutic targets for HCC. As HCC diagnosis and treatment status is not promising, many clinical trials looking for more ideal tools are underway. One of these tools is miRNA, which can be considered a promising HCC diagnostic and prognostic tool. The importance of miRNA dysregulation and expression has been confirmed in many cancers [31,32]. Chaotic miRNA expression is related to HCC tumorigenesis, and plasma miRNA expression has also been mentioned as a potential regulator of HCC [33].

Our previous study confirmed that miR-29a targets multiple biological function impacts, including attenuated shoulder stiffness fibrosis [34], liver fibrosis [8,12,35], and mitigated NAFLD [14,36], as well as influenced progression of HCC formation [26]. Similar findings have been confirmed by Song et al., who showed miR-29a downregulated Bcl-2 expression to ameliorate liver tumorigenesis [35]. In our previous study, we utilized and combined multiple public databases that perceived miR-29a expression was significantly decreased in HCC patients [16]. Of note, research establishes HIF-1α dominated roles in HCC patients that co-expression of HIF-1α and PD-L1 has significantly increased the risk of recurrence [37]. In this study, we also provide direct evidence that the two prognostic markers HIF-1α and ANGPT2 are upregulated in HCC patients and HCC animal models. In addition, our research also confirmed that miR-29a has a broader epigenetic effect on tumorigenesis-related gene expression and could regulate the expression of HIF-1α and ANGPT2 in HepG2 cells.

It is well-known that the microenvironment of tumorigenesis increased hypoxia status to promote neovasculogenesis for additional growth [38]. The significant role of HIF-1α in tumor development has been demonstrated in various tumor types and is responsible for tumor initiation, progression, and drug resistance [22]. Along with the crucial role of HCC growth under hypoxia conditions, the accumulation of reactive oxygen species (ROS) leads to the electron transport chain of mitochondrial obscurely and oxidative stress [39]. In this reaction, HIF-1α co-work with Notch signaling to modulate mitochondrial biogenesis metabolism and cross-reacted with HEY1 and PINK1 gene expression [39]. Furthermore, HIF-1α was also shown to strongly correlate with a higher rate of lymph node metastasis and vasculogenic mimicry [38].

Again, ANGPT2 (angiopoietin-2) as a prognostic marker plays a pivotal role in liver cancer and concomitantly with ANGPT1 (angiopoietin-1) and VEGF (vascular endothelial growth factor) promoted activity hypervascularity [17]. In steatohepatitis, the severity of pathogenesis was positively correlative with serum concentration of ANGPT2 and indispensable found, for which ANGPT2 excess of the HCC organization can be used as a predictor in HCC patients in recurrent or de novo rate. At the same time, the ANGPT2 showed a positive relationship with liver stiffness [40]. Evolving evidence suggests that ANGPT2 may utilize two molecular pathways to influence the growth of HCC and that ANGPT2 could secrete via exosomes and exist on the surface of HCC to promote epithelial-to-mesenchymal transition (EMT) activity [41]. On the other hand, the ANGPT2 and TIE2-expressing monocytes (TEMs) co-excess pathway has also been confirmed to have participated in metastatic and recurrent HCC [42].

Advanced HCC implicated multiple cellular pathways and played central roles in tumor metastasis and recurrence. Activity signaling cascade in HCC, including Ras/Raf/MEK/ERK and Ras/PI3K/Akt/mTOR, which promotes transcription of genes involved in tumor proliferation [43]. Sorafenib, a TKI (tyrosine kinases inhibitor) drug, was the first multi-kinase inhibitor authorized for the medicine of HCC. The efficacy of Sorafenib has been considered in numerous clinical trials and demonstrated affects many kinases, not only RAF and MEK, but other kinases, such as vascular endothelial growth factor receptor (VEGFR), platelet-derived growth factor receptor (PDGFR), and others. However, emerging studies found that advanced HCC patients who exhibit a B-RAS mutation or DCP (des-γ-carboxyl prothrombin) are more likely to be multifocal, aggressive, and resistant to TKI therapies [44,45]. Thus, finding more signaling pathways such as epigenetic moderators include Long non-coding RNA (lncRNA) [44], siRNA [46], miR agonists/antagonists [47], and small molecules [48], could help develop promising therapeutic strategies.

Trans-arterial chemoembolization (TACE) is a first-line treatment for patients with hepatocellular carcinoma (HCC) in Barcelona Clinic Liver Cancer stage B (BCLC-B) [2]. However, TACE puts hypoxic and chemotherapeutic stress on HCC, and some surviving tumors frequently transform into more aggressive and TACE-resistant tumor tissues [49,50]. As a result, hypoxia-induced by TACE can stimulate VEGF production by the residual tumor cells, promoting angiogenesis and ultimately tumor progression following TACE [51,52]. AMG386, a peptibody (first-in-class peptide-Fc fusion protein) that impedes ANGPT1/2 signaling, was exploited to develop trebananib for clinical studies with human patients [53,54]. Furthermore, HIF-1 activity is required to express some lysyl oxidase (LOX) family members, including Lox, Loxl2, and Loxl4 [55,56,57]. LOX family members are characterized by their catalytic activity contributing to structural integrity and increased tensile strength, acting to remodel the cross-linking of the structural extracellular matrix (ECM) of such fibrotic organs as the liver [58,59], as well as cancer microenvironments [58,60]. More recently, mounting evidence has recognized the emerging role of Lox and Loxl2/4 in fostering the corrupt microenvironment of HCC via angiogenesis promotion, epithelial-mesenchymal transition (EMT) program, and formation of pre-metastatic sites [55,61,62,63]. While the study delineates the contribution of miR-29a in the HCC development, considering the initial liver disease and the different stages of long-term development, which have increased the complexity of studying HCC. However, we are also aware of the experiment’s limitations that lack identification of miR-29a expression on human liver biopsies of various stages of HCC and engineering virus vector-mediated miR-29a to extend gene therapy strategies for HCC mice to explanation mechanisms on angiogenesis. In addition, the immune response is also the focus of future investigation. For instance, HIFs and miR-29a participated with liver tissue innate immunity [9,64]. In conclusion, profound evidence revealed that miR-29a loss, HIF-1α, and Angpt2 increase were correlated and pronounced influences with HCC development while contributing a clearly identifiable molecular mechanism in chronic liver disease.

## 4. Materials and Methods

### 4.1. miR-29a Interacted Cancer Gene Sets Platform Interpretation and Genes Differential Expression in HCC Patients Outcome Correlation

Bioinformatic predictive analysis of miR-29a regulate gene network in HCC patients, and candidate genes identification were performed public web analysis platforms, including OMCD (OncoMiR Cancer Database), UALCAN, GSCALite, TCGA (Cancer Genome Atlas), and TargetScan database. The detailed analysis procedure refers to the description of the previously published manuscript [15].

### 4.2. The HCC Mice Model Generative

The animal operative protocol, experimental procedures were reviewed and approved by the Institutional Animal Care and Use Committee (IACUC) of Kaohsiung Chang Gung Memorial Hospital (KCGMH, Affidavit No. 2020121109). Age 8 weeks C57BL/6N male mice (25–30 g) were purchased BioLASCO (Taipei, Taiwan), and all experimental animals were housed and followed IACUC use committee guidelines. Mice were randomized divided into two groups, which were fed with a regular chow diet (ND) or western diet (WD) containing 21.1% fat, 41% sucrose, and 1.25% cholesterol (TD. 120528, Teklad diets). Group of ND and WD were intraperitoneally given CCl4 (0.32 μg/g of body weight, 289116, Sigma-Aldrich, St. Louis, MO, USA) once a week for 25 weeks [65]. The animal measured body weight every four weeks until euthanasia and harvested the liver tissues for section and staining.

### 4.3. Liver Tissue Section and Staining

Tissue samples from at least two representative fragments of each liver lobes were taken and fixed in 10% paraformaldehyde for 36 h and, then, embedded in paraffin wax. Continuous 5 mm slices underwent Masson’s trichrome staining (Polysciences, NY, USA) in accordance with the manufacturer’s standard protocol and previously described [66]. For immunohistochemistry, tissue slides were dewaxed and conducted epitope retrieval (Thermal scientific at 95 °C for 30 min). Primary antibodies against Ki67 (ab15580, abcam, Cambridge, UK) were immunoreaction in sections to probed ki67 and reaction color use BioGenex detection kits (BioGenex, Fremont, CA, USA). Histological images were captured utilizing the digital slide scanner (Pannoramic MID), and images were randomly selected for quantification under constant magnification by ImageJ (V1.48) from three fields of each section and two sections of each liver specimen.

### 4.4. Cell Culture and Transfection

Human HCC cell line HepG2 purchased from American Type Tissue Collection (ATCC) and cultured in DMEM medium supplemented with 10% heat-inactivated fetal bovine serum (FBS), glutamax, and antibiotic-antimycotic at 37 °C in a humidified incubator with 5% CO_2_. HepG2 were seeded into 6-cm dishes (1.5 × 10^6^ cells/dish) overnight and then transfected with miR-29a precursor (a miR-29a mimic, GE Healthcare Dharmacon, Inc., Lafayette, CO, USA) or miR negative control (GE Healthcare Dharmacon, Inc.) for 24 h by using the Lipofectamine™ RNAiMAX Transfection Reagent (Invitrogen, Carlsbad, CA, USA), following the manufacturer’s instructions. The complete cell extracts are subjected to Western Blot analysis.

### 4.5. Quantitative RT-PCR

Total RNA in liver tissue was extracted by using TRIzol^®^ reagent (Invitrogen, CA, USA) and conducted reverse transcription of 1 μg total RNA to yield cDNA. The qPCR reaction was undertaken using 2× SYBR Green PCR Master Mix (04887352001, Roche Molecular Systems, Pleasanton, CA, USA) on LightCycler480^®^ (Roche). The specific primers for detected genes expression and detailed sequences of mouse genes are shown in Table 1.

### 4.6. Western Blotting

A total of 1.5 × 10^6^ cells were washed with PBS and lysed in protein lysis buffer (iNtRON Biotechnology, Seongnam, Korea), homogenized, centrifugated, and quantitative supernatant lysates (Bio-rad protein assay). Briefly, 25 μg protein extracts were employed and probed by primary antibodies of HIF-1α (Proteintech, 20960-1-AP), ANGPT2 (Proteintech, 24613-1-AP), and GAPDH (1:100,000; 60004-1-lg, Proteintech, IL, USA) as an internal control. Membranes were incubated with secondary antibodies against horseradish peroxidase-coupled anti-rabbit immunoglobulin-G antibodies (1:5000; NEF812001EA, PerkinElmer, Waltham, MA, USA) at room temperature for one hour. The signaling of blots was reacted with an ECL Western blotting system and exposed to film (GE Healthcare, Chicago, IL, USA). The signals were quantified using Quantity One^®^ 1-D analysis software (Bio-Rad Laboratories, Hercules, CA, USA). The accurate quantitative value of the target protein was normalized by its corresponding GAPDH.

### 4.7. Luciferase Reporter Activity Assay

The oligonucleotides that contained the HIF-1α or ANGPT 3′UTR target sequence were annealed and cloned into the pMIR-REPORTTM miRNA Expression Reporter Vector (Applied Biosystems, Waltham, MA, USA) to generate pMIR-HIF-1α luciferase plasmid or pMIR-ANGPT2 luciferase plasmid. The sequences in which the miR-29a binding site was replaced with the mutant site were annealed and cloned into the pMIR-REPORTM reporter vector to generate the pMIR-HIF-1α Mut luciferase plasmid or pMIR-ANGPT2-Mut luciferase plasmid. We then purified the plasmids using the EasyPrep EndoFree Maxi Plasmid Extraction Kit (BIOTOOLS, Ltd., Taipei, Taiwan). HepG2 cells were seeded at 3 × 10^6^ cells in a 10-cm dish for 18 h and transfected with a 6 μg reporter plasmid using Turbofect transfection reagent (Thermo Fisher Scientific, Rockford, IL, USA). After 18 h, the culture medium changed to a fresh medium and placed for 6 h. Post-transfection 24h, cells were trypsinized cells and seeded on a 6-cm dish with a density of 1.6 × 10^6^ cells/dish overnight. The cells were transfected with miR-29a precursor (a miR-29a mimic, GE Healthcare Dharmacon, Inc., Lafayette, CO, USA) or MIR negative control (GE Healthcare Dharmacon, Inc.) for 24 h using the Lipofectamine™ RNAiMAX Transfection Reagent (Invitrogen, Carlsbad, CA, USA) according to the manufacturer’s instructions. After 48 h transfection, cells were lysed for the detection of luciferase signal with Neolite Reporter Gene Assay System (PerkinElmer, Waltham, MA, USA) [67].

### 4.8. Statistical Analysis

The experiment results of transfected cells were repeated six times, and animal tissues as well and were presented as mean ± sem. Between two-groups compare was utilized *t*-test with unpaired two-tailed. Ordinary one-way ANOVA and Tukey’s multiple comparisons test with a single pooled variance were significantly analyzed for multiple groups. Statistical all values for significantly different as the *p* values were set at <0.05.

## Figures and Tables

**Table 1:** Sequence of primers pairs.

Gene Name	Forward Primers (5′➞3′)	Reverse Primers (5′➞3′)
*Col3a1*	ACGTAAGCACTGGTGGACAG	CAGGAGGGCCATAGCTGAAC
*Hif1a*	CGGAAACTCCAAAGCCACTT	GCTGGCTGATCTTGAATCTG G
*Angpt2*	CCGCGGGCAAAATAAGTAGC	CACATGCGTCAAACCACCAG
*Lox*	GACCACAGGGTACTGCTACG	TGGCTGAATTCGTCCATGCT
*Loxl2*	CTGACTTCCGCCCCAAGAAT	GTTGAGGCTCAGCAGGTCAT
*Vegfa*	CCCACGTCAGAGAGCAACAT	TGCGCTTTCGTTTTTGACCC
*Gapdh*	GCACAGTCAAGGCCGAGAAT	GCCTTCTCCATGGTGGTG

## Data Availability

The data that support the findings of this study are available on request from the corresponding author.
